# Strategic internal covalent cross-linking of TNF produces a stable TNF trimer with improved TNFR2 signaling

**DOI:** 10.1186/s40591-015-0044-4

**Published:** 2015-08-12

**Authors:** Liqin Ban, Willem Kuhtreiber, John Butterworth, Yoshiaki Okubo, Éva S. Vanamee, Denise L. Faustman

**Affiliations:** Immunobiology Laboratory, Harvard Medical School and Massachusetts General Hospital, Boston, MA USA

**Keywords:** TNF, Trimer, Cell signaling, TNFR2, Transmembrane TNF, Tumor necrosis factor, Tumor necrosis factor receptor

## Abstract

**Background:**

Soluble TNF superfamily (TNFSF) ligands are less stable and less active than their transmembrane (tm) analogues. This is a problem for the therapeutic use of recombinant TNFSF ligands in diverse diseases including cancer and autoimmunity. Creating TNFSF ligand analogues with improved targeting of their respective receptors is important for research and therapeutic purposes.

**Findings:**

Covalent internal cross-linking of TNF monomers by double mutations, S95C/G148C, results in stable trimers with improved TNFR2 function. The resulting mutein induced the selective death of autoreactive CD8 T cells in type-1 diabetic patients and demonstrates targeted proliferation and expansion of human CD4 Tregs.

**Conclusions:**

Stable TNF trimers, created by internal covalent cross-linking, show improved signaling. The high structural homology within the TNF superfamily provides an opportunity to extend internal cross-linking to other TNF superfamily proteins to produce active trimers with improved stability and receptor signaling, and with potential applications for cancer, autoimmunity, infections, and transplantation.

**Electronic supplementary material:**

The online version of this article (doi:10.1186/s40591-015-0044-4) contains supplementary material, which is available to authorized users.

## Findings

The tumor necrosis factor (TNF) superfamily, composed of both ligands and receptors, is one of the most successfully and frequently targeted protein families with clinical relevance to diverse diseases as cancer, autoimmunity, infections, graft rejection, inflammation and osteoporosis [1, 2]. Depending on the clinical situation, there is a need to both agonize and antagonize TNF superfamily (TNFSF) pathways. Most TNF superfamily (TNFSF) ligands are type II transmembrane proteins that act through more than one receptor, and like TNF can be cleaved to produce a soluble ligand that is less effective at signaling. All TNFSF ligands share the TNF homology domain and form non-covalent homotrimers [3]. Ligand trimerization is essential for signaling by TNFSF members. However, the trimers tend to dissociate at low concentrations such as in serum resulting in their degradation. Creating stable trimers of TNF and other TNFSF ligands is thus an essential step in generating functionally active receptor specific muteins [4, 5]. Various methods of secondary cross-linking of TNF ligands are also desirable but have faced challenges in increasing stability, half-life and improved signaling [6, 7]. Many TNF superfamily protein design approaches have been based on the construction of fusion proteins [7].

TNF is a pleiotropic cytokine with diverse functions in cell proliferation, inflammation, apoptosis and morphogenesis [1]. TNF is expressed as a tight trimeric transmembrane protein (tmTNF) that can be cleaved to produce soluble TNF (sTNF) [8, 9]. tmTNF and sTNF exert their functions via two receptors, TNFR1 and TNFR2. TNFR1 is ubiquitously expressed in the lymphoid system and in nearly all cells of the body, whereas TNFR2 has more limited cellular expression. sTNF binds with high affinity to both TNFR1 and TNFR2 but signals almost exclusively through TNFR1. Only tmTNF can effectively activate TNFR2 [10]. The picture that emerges from the literature indicates that the membrane serves at least two functions: 1) it anchors TNF, and increases its stability and bioavailability; and 2) it provides a platform for the arrangement of multiple TNF trimers for simultaneous signaling. The therapeutic use of recombinant sTNF has been hampered by its systemic toxicity and has been limited to localized limb and liver perfusions in certain cancers [11, 12]. TNFR2 agonism may provide a more targeted immunotherapy with reduced toxicity because of the more limited cellular expression of TNFR2. TNFR2 agonists and TNF-inducers have recently emerged as potentially new treatment strategies for specific and select autoimmune diseases such as multiple sclerosis and type-1 diabetes. TNF agonism, especially through the TNFR2 receptor in select autoimmune disease eliminates autoreactive T cells and induces beneficial T-regulatory cells [13–15].

Additional file 1: Methods description.

**Fig. 1:**
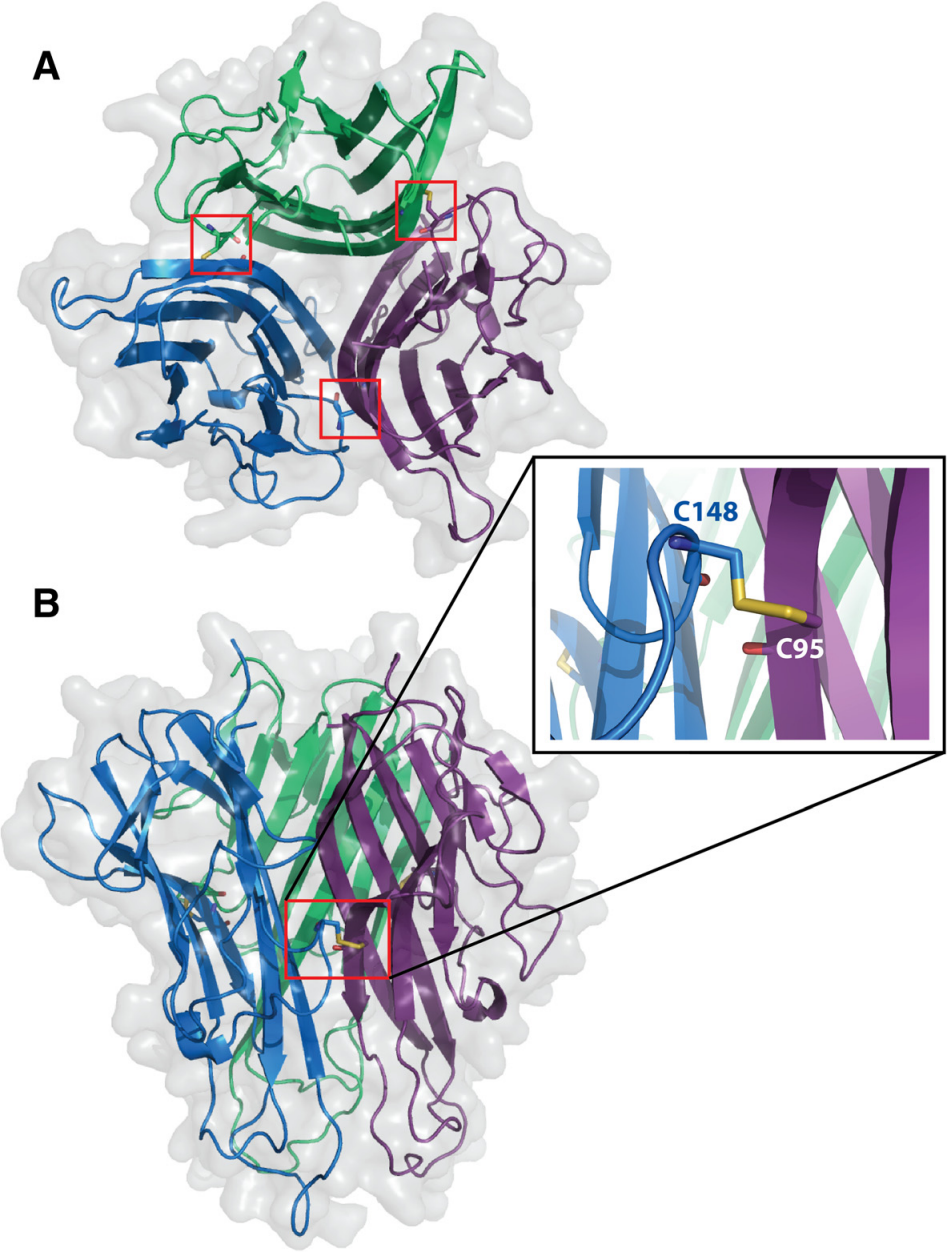
The 3D model of the covalently cross-linked TNF07 mutein. **a** Top view of the S95C/G148C double mutant. The three monomers are shown in cartoon representation and the monomers are colored in blue, purple and green. The surface representation is also shown in light grey. Red rectangles highlight the three Cys-Cys double bonds between each monomer. Residues 95 and 148 are shown in ball and stick representation. **b** Side view of the S95C/G148C double mutant. Red rectangle highlights one of the Cys-Cys double bonds and the insert shows the close up view. The model was generated by Modeller (v.9.12) [24] using the structure of mouse TNF (PDB ID: 2TNF) as the template. The pictures were created by the program PyMOL [25]

Building on our earlier research [13, 15], we set out to create sTNF muteins with TNFR2 agonism. As a first step, we created stable trimeric sTNF ligands by introducing an intermolecular Cys-Cys covalent bond. To our surprise, the S95C/G148C (TNF07) double mutant, in addition to being a stable trimer has also shown significantly improved TNFR2 signaling even without the addition of TNFR2 selective mutations. The double mutations in TNF07 (Fig. 1) were chosen after careful consideration of several factors. Ser95 sits on a structurally conserved beta strand and the isosteric Ser to Cys mutation is one of the most chemically conservative choices. Gly148 is found at the edge of loop 145–147 that participates in receptor binding but Gly148 itself is not involved in receptor interactions. A comparison of known sTNF structures shows that the position of residue 148 changes little between the free and receptor bound forms, maintaining at all times the ideal distance with residue 95 for S-S double bond formation. In addition, residues 95 and 148 are hidden from the surface (Fig. 1a) and are thus unlikely to induce immunogenicity or cause toxicity, unlike bulky external additions or chemical cross-linkers [16, 17].

**Fig. 2:**
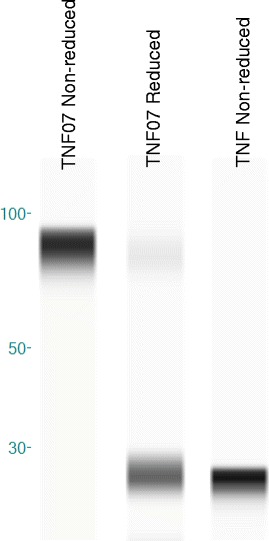
Analysis of TNF and mutein TNF07 on Western Blots under non-reduced and reduced conditions. Analysis on Western blot of TNF07 and sTNF under non-reducing conditions reveals sTNF (right lane) remains a monomer with approximate molecular weight (MW) of 25kD and the TNF07 (left lane) remains as a trimer with approximate MW of 75 kDa. With reduction, the stable TNF07 trimer becomes predominately a monomer with a 25kD MW (center lane)

Western blot analysis of the TNF07 mutein confirms a stable covalent trimer with an apparent molecular weight of 75 kDa compared to recombinant sTNF with an apparent molecular weight of 25 kDa (Fig. 2). Reduction of the trimer with dithiothreitol (DTT) resulted in the reappearance of the monomer confirming that trimerization is the result of disulfide cross-linking and not aggregation (Fig. 2). The stability of the TNF07 trimer was studied over a 3 year period. TNF07 muteins stored at −70 °C remained a stable trimeric structure with single thawing cycles and re-study on Western blots suggesting the S-S double bond formations were strong and stable. In comparison, recombinant sTNF is only stable for a few months after reconstitution and storage at −70 °C.

tmTNF is the preferred ligand for TNFR2 signaling. In contrast, sTNF poorly signals through TNFR2. Past work has shown murine and human Treg cells expand with TNFR2 agonism and the TNFR2 receptor is the controlling receptor for Treg expansion [15, 18, 19]. We studied the effects of the new TNF07 mutein in Treg expansion assays.

**Fig. 3:**
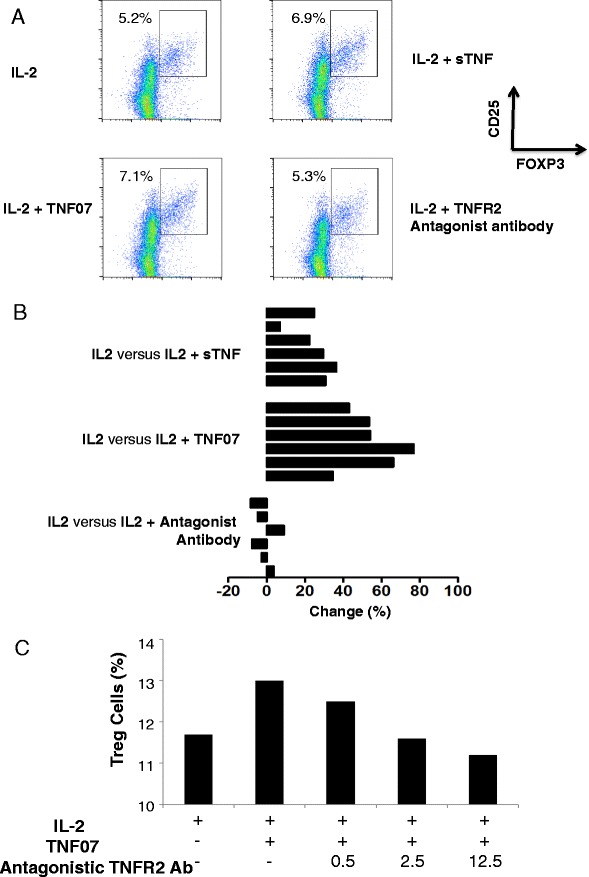
Functional traits of mutein TNF07 compared to sTNF and a TNFR2 antagonistic antibody in CD4 T-regulatory expansion assays. **a** Flow cytometry pictures of normal CD4 T cells treated for 16 h with IL-2, IL-2 plus sTNF, IL-2 plus TNF07 or IL-2 plus a TNFR2 antagonistic antibody. In this prototype experiment, isolated CD4 T cells with IL-2 incubation alone had a basal level of human T regulatory T cells (CD4 + CD25 + Foxp3+) of 5.2 % after the overnight incubation. In contract, treated with sTNF expanded the Tregs to 6.9 % and TNF07 expanded the Tregs more successfully to 7.1 %. In contrast, the same cells expanded with IL-2 plus a TNFR2 antibody antagonist showed halted Treg expansion (5.3 %). **b** The experiment is (**a**) was performed a total of 6 times on different CD4 human donors. Data is represented as the % increase or decrease in Treg cells with sTNF, TNF07 or with the TNFR2 antagonistic antibody compared to IL-2 alone overnight incubations. A = IL2 vs IL2 + Antagonist Ab (*p* < 0.001), B = IL2 vs IL2 + TNF-07 (*p* = 0.0003), C = IL2 vs IL2 + sTNF (*p* = 0.0027). **c** An inhibition experiment shows the ability of a monoclonal TNFR2 antagonistic antibody with dose escalations (ug/ml) to prevent the agonism of the TNF07 mutein

In a set of representative flow cytometer pictures from a single donor, Treg expansion with the new TNF07 mutein was 7.1 %, with sTNF was 6.9 % or to Il-2 alone at 5.2 % (Fig. 3a). The TNF07 mutein appeared superior to sTNF that functions preferentially through TNFR1. As a control experiment, the same isolated CD4 T cells were also treated with TNFR2 antagonistic antibodies with resulting Treg static counts (5.3 %). This experiment was repeated on 6 additional control CD4 cell samples and statistically significant differences between the groups were observed (Fig. 2b). Each time the sTNF exposures to isolated CD4 T cells showed some Treg expansion but each time the TNF07 mutein was superior at Treg expansion, a feature of TNFR2 preferential utilization.

To further demonstrate the improved TNFR2 activity of TNF07, a competitive dose response experiment was set up with escalating doses of a TNFR2 antagonistic antibody. The data clearly shows that addition of a TNFR2 antagonistic antibody in a dose response fashion blocked TNFR2 agonism and Treg expansion (Fig. 3c).

**Fig. 4:**
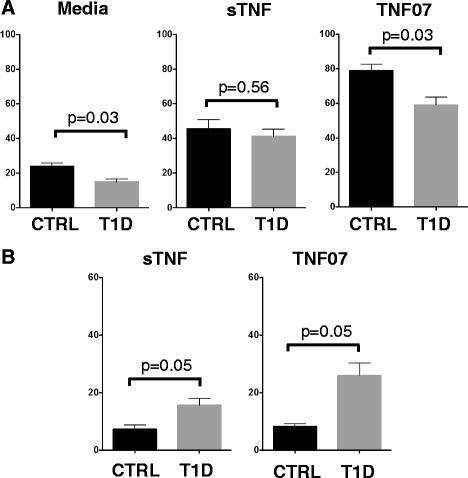
Proliferation or death of CD8 T cells from type 1 diabetics or controls with sTNF or TNF07 compared to media. The WST-1 cell proliferative assay or the LDH cell death assay were used to study the effects of TNF or the TNF07 mutein on type 1 diabetic (T1D) or control (CTRL) CD8 T cells. **a**. Data shows that CD8 T cell proliferation measured in the WST-1 assays was greater with either TNF and even greater for TNF07 treated CD8 T cells compared to media alone. Also for each T1D compared to control CD8 T cells the diabetic proliferated was less: this was most pronounced with the TNF07 mutein. **b**. In an opposite fashion, cell death measure by LDH assay shows that CD8 diabetic T cells compared to control T cells were more susceptible to death, especially with TNF07 over sTNF. TNF07 induced death was greater in the type 1 diabetic (*p* = 0.05). Overall CD8 T cell proliferation, a trait of agonism of the TNFR2 receptor was greater for TNF07 than TNF and cell death of CD8 autoreactive T cells, a process driven by CD8 TNFR2 agonism is more pronounced in the diabetic. The data presents 10 normal donors and six type 1 diabetics

TNFR2 agonism is an improved proliferative signal for overall CD8 T cell expansion over TNFR1 or sTNF signaling. TNFR2 agonism also promotes selective autoreactive CD8 T cell directed death in type 1 diabetic cells [13]. We set out in CD8 proliferative (WST-1) and cell death (LDH release) assays to functionally test if TNF07 preferentially target TNFR2. In the WST-1 proliferation assay both control and diabetic CD8 T cells expanded slightly with media supplemented with IL2 overnight although the control proliferation was slightly greater (24+/−2.1 versus 15 +/− 1.7; *p* = 0.03) (Fig. 4a). The addition of sTNF equally induced greater proliferation of control and diabetic CD8 T cells over media (46+/−5.4 versus 41+/−4.1, *p* = 0.56) (Fig. 4a). In marked contrast, the application of TNF07, a punitive TNFR2 specific agonist, to control and type 1 diabetic cells caused marked CD8 expansion; again the control proliferation was slightly greater than the type 1 diabetic proliferation (79 +/−3.8 versus 59+/−4.6, *p* = 0.03) (Fig. 4a). This proliferation data suggested functionally TNF07 was acting as a TNFR2 agonist for CD8 proliferation.

To further test whether TNF07 was acting as a functional TNFR2 agonist, we sought evidence that the less robust proliferation of CD8 T cells with TNF or TNF07 in Fig. 4a was due to TNF07 induced autoreactive T cell death in the diabetic through TNFR2 agonism [13, 15]. It was suspected that TNF or even better the TNF07 mutein was killing a minor subpopulation of autoreactive CD8 T cells in the culture of the type 1 diabetic cells. To prove this, isolated CD8 T cells were studied in the LDH cell death assay. In normal donors, CD8 T cells showed background cell death with either sTNF or TNF07 (7.3+/−1.5, 8.3+/−0.9, respectively) (Fig. 4b). In contrast, selective death of autoreactive CD8 T cells of type 1 diabetic T cells could be observed with sTNF and even more pronounced with TNF07 (Fig. 4b). This cell death assay again points to TNF07 showing preferential TNFR2 signaling.

Here we show that the covalent cross-linking of sTNF at a strategic location between TNF monomers can generate a stable trimer with improved functional agonistic consequences on human CD4 or CD8 T cells. The results of the human functional T cell assays indicate that TNF07 has improved TNFR2 agonism, more potent than sTNF. Internal cross-linking at a different location did not result in improved TNFR2 activity. TNF07 not only induced the selective death of autoreactive CD8 T cells in type-1 diabetic patients, it also demonstrated impressive proliferation of normal CD8 T cells. TNF07 was also capable of expanding CD4-Treg cells, again a function of TNFR2 agonism. Various defects in TNF signaling pathways occur in both human and mouse models of several autoimmune disorders. sTNF, TNF inducers or TNFR2 agonism have shown promise for treating these subtypes of autoimmune diseases [20]. Due to the more limited expression of TNFR2, TNFR2 agonism provides a potentially more targeted therapy with fewer side effects. Another TNF construct referred to as TNFR2-specific sc-TNF variant has been shown to rescue human neurons from cell death highlighting the potential application of TNF analogues, especially with preferential TNFR2 agonism in neurodegenerative diseases [21]. TNFR2 agonism is also known to facilitate diverse forms of end organ repair [22]. TNF07 was not designed to have TNFR2 selectivity. Future efforts will focus on removing TNFR1 activity to reduce systemic toxicity for potential therapeutic applications.

Due to the high structural homology, the concept of disulfide cross-linking is broadly applicable to all TNFSF ligands. It provides an alternative to other trimer stabilizing methods as a more naturally appearing, and less immunogenic structure upon human exposure. It is an expandable system that can be combined with receptor specific mutations to selectively target various TNFSF receptors, and also with secondary cross-linking methods of either the TNFSF ligands or their receptors [6, 7, 23].

## Additional file

Additional file 1:
**Methods description.** (DOCX 102 kb)

## Abbreviations

sTNF: soluble Tumor necrosis factor
tmTNF: transmembrane TNF
Tregs: T regulatory cells
TNFR2: TNF-receptor 2
TNFSF: TNF superfamily
T1D: type 1 diabetic
CTRL: Control
