# Supplementary material for: Strategic internal covalent cross-linking of TNF produces a stable TNF trimer with improved TNFR2 signaling
Source: Mol Cell Ther. 2015 Aug 12;3:7. doi: 10.1186/s40591-015-0044-4 (PMC4531505; doi:10.1186/s40591-015-0044-4)
Supplement: Additional file 1: — Methods description. (DOCX 102 kb) [file 40591_2015_44_MOESM1_ESM.docx]

**Additional file1: Methods description.**

**Materials and Methods**

**Human Subjects**

Subjects used in this study were either type 1 diabetics or non-affected control subjects. They were recruited from Massachusetts General Hospital with full institutional approval and informed consent (MGH-2001P001379). Each subject donated 4 tubes of blood and the blood was used fresh in the morning for the preparation of either CD8 T cells for the WST-1 proliferation/death assay or for CD4 T cells for the T regulatory assays. For the WST-1 assays of cell proliferation or death, each autoimmune subject was simultaneously studied with a control subject. All blood was drawn into BD Vacutainer tubes containing acid citrate and dextrose.

**Blood preparation**

Purified CD4 or CD8 T cell subsets were isolated from peripheral blood lymphocytes using Dynal magnetic isolation methods (Invitrogen: Product Nos 113-33D and 113-31D). This method yielded fresh human CD4 or CD8 T cells that were over 95% viable. PBLs were isolated with Ficoll-Hypaque gradients. These methods have been previously described [1, 2]

**Construction of TNF and the TNF mutein**

Construction of TNF and the mutein TNF07 involved original gene synthesis of the DNA fragments into the His6-thrombin-site-TNF gene-fusion and these were subcloned into pDEST42 expression vector. Positive clones were confirmed by restriction enzyme digestion and sequence validation. TNF and the muteins were expressed in E. coli strain BL21(DE3)pLysS. A seed culture was incubated at 37^⁰^C for 16 hours and was used to inoculate 4 liter of tissue culture media which was incubated at 37^⁰^C until A600=0.8. Protein expression was induced with IPTG (0.1mM) for 16 hours at 18^⁰^C. Cells were harvested by centrifugation and stored at -80^⁰^C.

Cell paste was suspended in Buffer A (20 mM Tris, pH 7.9, 500 mM NaCl, 5 mM Imidazole, 0.01% NP-40) and lysed using a PANDA homogenizer. The lysate was clarified by centrifugation and the supernatant was applied to a 5-ml Ni-NTA sepharose column (Qiagen) using a AKTA Explorer (GE Healthcare). The column was washed with 10 column columns Buffer A and the bound protein was eluted with a linear gradient of 0-100% Buffer B collecting 5ml fractions. Aliquots of protein containing fractions were mixed with SDS-sample buffer and electrophoresed under reducing conditions on 4-20% gels. Gels were stained with Coomassie Brillant Blue (R-25). In the Western blot analysis, anti-his antibodies (Clontech) were used.

The N-terminal polyhistidine-tag was cleaved using a Thrombin cleavage capture kit (Novagen, Cat# 69022-3) following the manufacturer’s protocol. Biotinylated thrombin was removed from the protein prep using streptavidin agarose following the manufacturer’s protocol.

Protein concentration was determined by Bradford’s method. The final pool was buffer-exchanged into 1X PBS, 0.1% albumin (pH 7.4) snap frozen in 0.5 ml aliquot and stored at -80⁰C. The final protein prep was subjected to N-terminal sequencing to confirm protein identity and correct His-tag removal. The model was generated by Modeller (v. 9.12) using the structure of mouse TNF (PDB ID: 2TNF) as the template. The pictures were created by the program PyMol.

**T-regulatory assay**

CD4 T cells were isolated from fresh human blood within 2 hours of venipuncture using Dynal CD4 Positive Isolation Kit (Invitrogen). We modified the protocol recommended by the manufacturer by using HBSS supplemented with 2% fetal bovine serum (FBS) (Hyclone, Logan, UT) instead of PBS. To verify the quality of isolated cells, they were assessed to be greater than 98% in purity and 96% in viability by CD4 and propidium iodide staining. After isolation, 2 x 10^5^ cells were plated in 96 round-bottom well and treated with TNF (20ng/ml) or TNF07 (20ng/ml) or a TNFR2 antagonist antibody from the Immunobiology Core (Harvard Medical School, Massachusetts General Hospital, Boston, MA). In some experiments IL-2 (50U/ml) was added. After 16 hours, cells were collected and intracellular FOXP3 was determined by flow cytometry. These methods have been previously described [3].

**Cytotoxicity/Proliferation Assay**

The WST-1 (Roche Applied Science) assay was used to proliferation. The LDH assay was used to confirm cell death (Pierce/Life Technologies). Each assay was used in combination with CD8 cell in media, sTNF or with the TNF07 mutein. The CD8 cells were isolated from type 1 diabetics (T1D) or from control (CTRL) individuals.

The WST-1 assay is a cell proliferation assay. The isolated CD8 T cells were plated into 96 well U-bottom plates with a cell concentration of 100,000 cells/well. Cells were cultured overnight at 26^⁰^C in RPMI media with 1% heat inactivated fetal calf serum (FCS). In the morning, the cells were treated with TNF or the TNF mutein for 1 hour. After the 1-hour exposure to TNF or the TNF mutein, the WST-1 reagent (44-[3-(4-iodophenyl)-2-(4-nitrophenyl) 2H-5-tetrazolio]-1,3-benzendisulfonate) was added according to the manufacturer’s instructions. The cleavage of WST-1 to fromazan by metabolically active cells was quantified by Beckman Coulter DTX 880 Spectrophotometer (Beckman Coulter) at a wavelength 405 m. Each experiment was performed in triplicate. Test medium was used as background control. The cells treated with various doses of ligand are presented as a percentage of proliferation compared to the untreated cells using the following equation: TNF or TNF mutein treated – untreated/untreated.

**Reagents**

Recombinant human and mouse TNF was purchased from Leinco Technologies (St. Louis, MO), and recombinant human IL-2 was purchased from Sigma-Aldrich (St. Louis, MO). Human monoclonal antibodies against TNFR2 were from internal sources. Intracellular staining of FOXP3 and CD25 were performed using either FOXP3 Fix/Perm Buffer set (Biolegend) or Human FOXP3 Buffer set (BD Biosciences).

**Western blot**

TNF and mutein TNF07 were analyzed on a Peggy Automated Western Assay Platform in size separation mode (ProteinSimple, Santa Clara, CA). Samples (0.5 ug/uL) were mixed at a ratio of 3:1 with a 4x Mastermix containing fluorescent molecular weight standards and sample buffer, with or without DTT (Dithiothreitol) reducing agent. To further prevent reduction of the non-reduced samples, the samples were not subjected to high (95°C) temperature as is customary, but rather were kept on ice until use. All running parameters were left at their defaults, except for loading time (12 seconds instead of default of 8 seconds) and for first antibody incubation time (240 min instead of default of 120 min). Detection of TNF was performed with a monoclonal antibody to human TNF (Abcam ab8348, Cambridge, MA) followed by HRP-labeled second antibody and Luminol/H_2_O_2_.

**References**

1. Ban L, Zhang J, Wang L, Kuhtreiber W, Burger D, Faustman DL: **Selective death of autoreactive T cells in human diabetes by TNF or TNF receptor 2 agonism**. *Proc Natl Acad Sci U S A* 2008, **105**:13644-9

2. Faustman DL, Wang L, Okubo Y, Burger D, Ban L, Man G et al.: **Proof-of-concept, randomized, controlled clinical trial of Bacillus-Calmette-Guerin for treatment of long-term type 1 diabetes**. *PLoS One* 2012, **7**:e41756

3. Okubo Y, Mera T, Wang L, Faustman DL: **Homogeneous expansion of human T-regulatory cells via tumor necrosis factor receptor 2**. *Sci Rep* 2013, **3**:3153
